# Supplementary material for: Patterns of Intron Gain and Loss in Fungi
Source: PLoS Biol. 2004 Nov 30;2(12):e422. doi: 10.1371/journal.pbio.0020422 (PMC532390; doi:10.1371/journal.pbio.0020422)
Supplement: Table S1 — Also available at http://genes.mit.edu/NielsenEtAl/. (4.3 MB ZIP). [file pbio.0020422.st001.zip › NielsenEtAl/html/459.html]

AN0385.1.NCU00792.1.MG02125.1.FG05696.1


```
 CLUSTAL W (1.82) Multiple Sequence Alignments - Introns Inserted


Sequence 1: NCU00792.1	413 aa
Sequence 2: MG02125.1	386 aa
Sequence 3: FG05696.1	380 aa
Sequence 4: AN0385.1	394 aa
Alignment Length: 421 aa
Number Identitical Residues: 196 aa
Alignment Score (without introns) 9279


MG02125.1 	-MASSFPPEPVSTIDWSNVGFKIREV1NGHIESHYSVQTGEWSPLKWVEDPYIRIHGMAP
NCU00792.1	-MAN-FPPPPVSTIDWSNVGFKVREV1NGHIESSYSVKTGKWSPLKFVSDPYLRMHGMAP
FG05696.1 	--MTAFPPPPVNTIDWSNVGFKVREV1NGHVESTYSKITGKWTPLRFVADPFMRIHGMAP
AN0385.1  	MASQSFPPPPVDTIDWSNIGFKVREV1NGHVESHYTPATKSWSPPKLVKSPYLPIHGMAP
          	    :*** **.******:***:*** ***:** *:  * .*:* : * .*:: :*****

MG02125.1 	ALNYGVQAYEGLKAFRGPGDKTIQIFRPDRNATRLQHSASFICIPPVPVQLFMDAVTAAV
NCU00792.1	ALNYGQQAYEGLKAFRLPGNDSIAIFRADKNASRMQHSAEYISIPPVPVDLFIDAVKAAV
FG05696.1 	ALNYGQQAYEGLKAFRTPNDEAITVFRPDRNAKRLQHSAEVVSMPPVPEDLFLSAVRAAV
AN0385.1  	GLNYGQQAYEGLKAFRHPNNSKITIFRPDRNALRMQRSASFISIPPVPEDLFLEAVELAV
          	.**** ********** *.:. * :**.*:** *:*:**. :.:**** :**:.**  **

MG02125.1 	ARNAEYVPPHETGAAMYIRPQIYGSSAQLGLNPCEEYTFAVYVVPTGVYHGTAPTKAFIL
NCU00792.1	ALNAEFVPPHETGAALYIRPQIFGSSAQLGLSPPEEYLFSVFVIPTGVYHGTHPVKALIL
FG05696.1 	ALNAEYVPPHDTGAALYIRPQLYGSSAQLGLSPPDEYIFAVFVIPTGVYHGAHPVKALIL
AN0385.1  	GANAGFVPPHETGAAMYIRPLIFGSSAQLGLSPPEEYTFVVFVMPTGVYHGVHAVDALIL
          	. ** :****:****:**** ::********.* :** * *:*:*******. ...*:**

MG02125.1 	DEFDRAAPNGTGSAKVGGNYAPVLKWGEKARSEGYGINLHLDSATHSEIDEFSTSGFIGA
NCU00792.1	EEFDRAAPNGTGSAKVGGNYAPVLRWSDKARKEGYDITLHLDSARHEEVDEFSTSGFIGV
FG05696.1 	EDFDRAAPHGTGNAKVGGNYAPVLRWSDKARDEGYGITLHLDSVRHEEIDEFSTSGFIGV
AN0385.1  	EDFDRAAPEGTGSAKVGGNYAPVLRHSAKAHAEGFGITLHLDSRTRSEIDEFSTSGMIAV
          	::******.***.***********: . **: **:.*.*****  :.*:*******:*..

MG02125.1 	I--DDGQGNITIVVPDSKNVIDSVTSESIQEIG-ASFGWKVEKRS0IKYTELPTFSEVMA
NCU00792.1	LRGDGGEDDVTVVVPSSKAVIDSVTSDSIQEIG-RSFGWKVEKRP0IKYSELPSFSEVMA
FG05696.1 	K---VNGDDVTLSVPDSKNVIDSVTSDSIQELG-RSYGWKVEKRA~IKYGELKEFSEVFA
AN0385.1  	KK-NKESGKVTLVQPDSPNVIDSVTAASVCEIGKLWFGYDVEKRR~IPYEELNEFDEVMA
          	   .   ..:*:  *.*  ******: *: *:*.  :*:.****  * * **  *.**:*

MG02125.1 	AGTAASLVPIRSITRKISSSDPKSLAAT--VKQHPRLEVT--VDQETVTYVPSSQPEAG-
NCU00792.1	AGTAAALVPIRSITRRVKTSSPQSLASSTATQEHARVSFNKEAEEETVTYLPDNQEDAG-
FG05696.1 	AGTAAALVPIRSITRRVDG------------------------KEDIATYIKEGSEEPG-
AN0385.1  	AGTAAALVPIRSITRRSSG--------------------------NRFEYECGGEEAGGG
          	*****:*********: .                           :   *   ..   *.

MG02125.1 	PICTKLLSQFKGIQLGKVKDEFGWNHMVTEADTKKAA-----------------------
NCU00792.1	DLCLKLLSQLKGIQLGKIEDKLGWRFAVTEEDGKKAEGAEKARSSEGQGKEAQTVDQMD-
FG05696.1 	PLFEKLLKHLKDIQLGRAEDSFSWRFPVGVKDKEIQG------ASTGGNGDATTVDQMD-
AN0385.1  	EVCVKLLRTLKGIQLGKIEETLGWNRVVKAPPAEWVGAADEKEEAGIEVPQACRMICTAS
          	 :  ***  :*.****: :: :.*.  *     :   .:..   :     .:       :

MG02125.1 	---
NCU00792.1	---
FG05696.1 	---
AN0385.1  	NWY
          	.
```
